# General practice and patient characteristics associated with personal continuity: a mixed-methods study

**DOI:** 10.3399/BJGP.2022.0038

**Published:** 2022-10-04

**Authors:** Marije T te Winkel, Pauline Slottje, Anja JTCM de Kruif, Birgit I Lissenberg-Witte, Rob J van Marum, Henk J Schers, Annemarie A Uijen, Jettie Bont, Otto R Maarsingh

**Affiliations:** Department of General Practice, Amsterdam UMC, Vrije Universiteit Amsterdam, Amsterdam; Aging and Later Life, Amsterdam Public Health, Amsterdam.; Department of General Practice, Amsterdam UMC, Vrije Universiteit Amsterdam, Amsterdam; Quality of Care, Amsterdam Public Health, Amsterdam.; Department of Epidemiology and Biosciences, Amsterdam UMC, Vrije Universiteit Amsterdam, Amsterdam; Health Behaviors and Chronic Diseases, Amsterdam Public Health, Amsterdam; Department of Nutrition, Dietetics and Lifestyle, School of Allied Health, HAN University of Applied Sciences, Nijmegen.; Department of Epidemiology and Biostatistics, Amsterdam UMC, Vrije Universiteit Amsterdam, Amsterdam; Methodology, Amsterdam Public Health, Amsterdam.; Department of Medicine for Older People, Amsterdam UMC, Vrije Universiteit Amsterdam, Amsterdam; Aging and Later Life, Amsterdam Public Health, Amsterdam.; Department of Primary and Community Care, Radboud University Nijmegen Medical Centre, Nijmegen.; Department of Primary and Community Care, Radboud University Nijmegen Medical Centre, Nijmegen.; Department of General Practice, Amsterdam UMC, University of Amsterdam, Amsterdam; Quality of Care, Amsterdam Public Health, Amsterdam.; Department of General Practice, Amsterdam UMC, Vrije Universiteit Amsterdam, Amsterdam; Aging and Later Life, Amsterdam Public Health, Amsterdam.

**Keywords:** continuity of patient care, general practice, personal continuity, primary health care, mixed methods

## Abstract

**Background:**

Personal continuity of care is a core value of general practice. It is increasingly threatened by societal and healthcare changes.

**Aim:**

To investigate the association between personal continuity and both practice and patient characteristics; and to incorporate GPs’ views to enrich and validate the quantitative findings.

**Design and setting:**

A mixed-methods study based on observational, routinely collected healthcare data from 269 478 patients from 48 Dutch general practices (2013–2018) and interviews with selected GPs.

**Method:**

First, four different personal continuity outcome measures were calculated relating to eight practice and 12 patient characteristics using multilevel linear regression analyses. Second, a thematic analysis was performed of semi-structured interviews with 10 GPs to include their views on factors contributing to personal (dis) continuity. These GPs worked at the 10 practices with the largest difference between calculated and model-estimated personal continuity.

**Results:**

Both a larger number of usual GPs working in a practice and a larger percentage of patient contacts with locum GPs were dose-dependently associated with lower personal continuity (highest versus lowest quartile −0.094 and −0.092, respectively, *P*<0.001), whereas days since registration with the general practice was dose-dependently associated with higher personal continuity (highest versus lowest quartile +0.017, *P*<0.001). Older age, number of chronic conditions, and contacts were also associated with higher personal continuity. The in-depth interviews identified three key themes affecting personal continuity: team composition, practice organisation, and the personal views of the GPs.

**Conclusion:**

Personal continuity is associated with practice and patient characteristics. The dose-dependent associations suggest a causal relationship and, complemented by GPs’ views, may provide practical targets to improve personal continuity directly.

## INTRODUCTION

Personal continuity of care is considered one of the core values of general practice.^1^^–^5 Personal continuity implies familiarity and mutual confidence between patient and doctor that can, and usually does, arise from repeated contacts over time.^6^ Reported benefits include a better patient–doctor relationship,^7^^,^^8^ better preventive care,^9^ fewer emergency department visits,^10^ greater patient and doctor satisfaction,^8^^,^^11^^,^^12^ fewer hospital admissions,^13^ reduced healthcare costs,^14^ better medication compliance and use,^9^^,^^15^^–^19 and reduced mortality rates.^20^^,^^21^ Adverse effects of personal continuity include frustrated or difficult patient–doctor relationships, and delayed diagnosis or referrals.^22^

Sandvik *et al* found an association between the length of the relationship between a patient and their usual GP and lower use of out-of-hours services, fewer acute admissions to hospital, and lower mortality.^23^ These associations were dose-dependent and probably causative, suggesting that any improvement in personal continuity may influence these outcomes and benefit the patient.

However, societal and healthcare changes potentially reduce personal continuity. For example, GPs increasingly work part-time and in larger practices.^1^^,^^2^^,^^5^^,^^24^ Both patients and doctors are increasingly mobile.^1^ The prevalence of complex, chronic diseases is increasing.^1^ Finally, patients increasingly expect fast access to any doctor.^2^^,^^3^ Together, these changes result in fragmented care from different providers, organisations, and disciplines. In addition, high workload levels and workforce shortages could limit GPs’ ability to realise personal continuity.^25^ Consequently, in recent years, personal continuity has declined in general practice.^26^

**Table:** How this fits in

Personal continuity of care, a core value of general practice, is threatened by societal and healthcare changes. To identify practice and patient characteristics associated with personal continuity, this study used both observational routinely collected care data and semi-structured interviews with GPs. The dose-dependently associated characteristics (that is number of usual GPs in a practice and days since registration with a practice), combined with GPs’ views, may provide practical targets for future interventions to improve personal continuity in general practice.

In order to optimise personal continuity, it is important to identify practice and patient characteristics that are associated with discontinuity.^27^ Patient characteristics could help to determine patients who are prone to discontinuity, whereas associated practice characteristics may enable practices to identify organisational factors that promote or obstruct personal continuity.^27^ For example, previous research from Canada, Norway, and the UK has shown that older patients are more likely to see their usual doctor.^27^^–^29 If practices offered patients a convenient appointment system, patients were more likely to have contact with their preferred GP.^26^ Other factors that may influence personal continuity include age, sex, ethnicity, income, education, and patient preferences.^30^ In addition, practices with larger list sizes (>6000 patients) had lower personal continuity than practices with smaller list sizes.^27^^,^^31^ One study showed that personal continuity was inversely associated with the number of GP leave days and being a training practice.^32^ In contrast, rurality and percentage of scheduled appointments with an assigned healthcare provider were positively associated with personal continuity.

However, to the authors’ knowledge, only one study exists on the association between personal continuity and both practice and patient characteristics in general practice.^27^ Therefore, in the current study both practice and patient characteristics and established outcome measures were incorporated, which were complemented by including GPs’ views. The aims of the study were to:

- investigate the association between personal continuity and both practice and patient characteristics; and
- incorporate GPs’ views to enrich and validate the quantitative findings.

## METHOD

### Study design

A mixed-methods approach to studying the association between personal continuity and practice and patient characteristics was used. First, observational, routinely collected health data from 48 general practices associated with the Academic Network of General Practice at Amsterdam University Medical Center, located at VU University Medical Center (ANH VUmc), was analysed. Second, 10 semi-structured telephone interviews were conducted with 10 GPs from different practices using a purposive sampling strategy.

### Quantitative methods

#### Data collection, access, and cleaning methods

**Figure 1.**
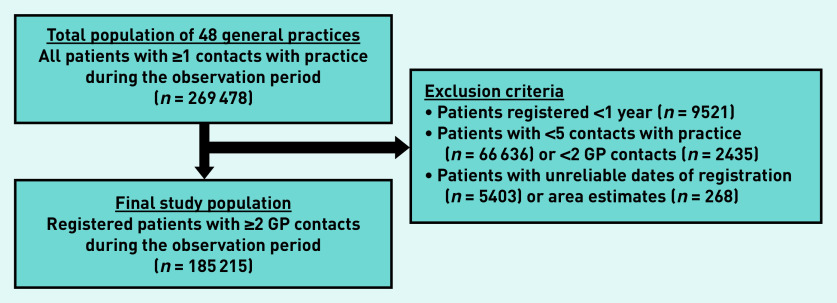
*Selection of the final study population. Area estimate = estimates based on areas with the same zip code. GP contacts = contacts with a GP.*

In total, the 48 practices included in this study provided care for 269 478 patients in a 6-year observation period (2013–2018). All non-institutionalised citizens (patients who are not residing in a hospital or other institution, for example, psychiatric home) are registered with one general practice.^33^ These practices provide care during office hours.^33^ Initially, patients with at least one contact with their general practice were selected as potential participants in this study. In order to gather meaningful data and be able to calculate personal continuity, the authors of this study reached consensus about selecting patients who were registered for ≥1 year; and had ≥5 contacts with their practice, including ≥2 with a GP between 2013 and 2018 (Figure 1). Three of the authors had access to the anonymised analytical dataset.

#### Continuity measures (dependent variable)

To calculate personal continuity between GP and patient, telephone calls, home visits, emails, and face-to-face consultations that were registered in the electronic medical record by a GP were included. For each patient, included GP contacts were used to calculate four established continuity outcome measures:

- Usual Provider of Care (UPC);
- the Herfindahl–Hirschman Index (HI);
- the Continuity of Care Index, also known as the Bice–Boxerman Index (BBI); and
- the Modified Modified Continuity Index (MMCI).^34^^–^37

All the continuity outcome measures have ranges between 0 (minimal, that is, all contacts are with different GPs) and 1 (maximal, that is, all contacts are with the same GP). Practice continuity was determined by aggregating continuity outcomes for its individual patients.^5^ In the main text, the results of the continuity outcome measure with the highest explained variance (MMCI) is provided. For calculations see Supplementary Table S1.

### Determinants

#### Practice characteristics

Based on suggestions from the literature and availability in the data, the ANH VUmc collected practice and patient characteristics for each practice. The current study then included the number and working hours of the usual GPs, number of usual GPs working >5 years at the practice, number of locum GPs, and percentage of contacts with locum GPs (between 2013 and 2018).^2^^,^^29^^,^^35^^,^^38^ Usual GP is defined as a partner or salaried GP who usually works at this practice.^27^ Other practice characteristics included list size,^2^^,^^27^ training practice,^39^ and number of other employees.^30^

#### Patient characteristics

For each individual patient, the sociodemographic variables sex, age, and an estimate of income and migration background, were included.^30^ The estimates were provided by ANH VUmc data managers, based on the patients’ 4-digit zip code data (1 January 2016) and data from the National Statistical Office (Statistics Netherlands).^40^ The local median income (low, average, or high), corrected for differences between family compositions, was provided by the National Statistical Office. Similarly, migration background was based on the local percentage of the population who, or whose parents, were born in Africa, South America, Asia (excluding Indonesia and Japan), or Turkey.

Information on the patient’s medical history was also included relating to chronic diseases, and specifically coronary heart, oncological, and psychiatric diseases.^30^^,^^41^^–^43 These diagnoses are registered by Dutch GPs using the International Classification of Primary Care, version 1 (ICPC-1 NL), and defined by the Netherlands Institute for Health Services Research (see Supplementary Tables S2a and S2b for details).^44^^,^^45^ Additionally, the number of days since registration at that practice, the number of contacts, and percentage of telephone calls and home visits were calculated.^30^

### Quantitative analysis

All statistical analyses were conducted on the final dataset with IBM SPSS Statistics (version 26). Data were summarised with mean and standard deviation (SD) or median with ranges for normally distributed and non-normally distributed continuous characteristics, respectively. Categorical patient characteristics were described with the mean and SD percentage per practice.

To determine the association between personal continuity and the characteristics, linear mixed models were built with fixed effects for patients and practice characteristics, and random intercepts for patients nested within practices. First, a backward selection procedure was used to identify the statistically significant patient characteristics by removing those characteristics with the highest *P*-value one-by-one until all remaining had *P*≤0.001. Second, the same procedure was used to identify the remaining associated practice characteristics. Continuous practice characteristics were categorised into quartiles because plots showed that the linearity assumption was not met.

Models were estimated for each of the continuity measures separately, to determine the best fit. The quality of the models was measured by the amount of explained variance of the models for both practice and patient characteristics. To determine the additional value of the practice characteristics, the explained variance of the model for patient characteristics only was also calculated. Additionally, likelihood ratio tests were conducted to confirm the best version of the model for each of the continuity outcomes. The model for the continuity outcome with the highest explained variance was considered the best fit and displayed in the results (other outcomes can be found in Supplementary Tables S3 [descriptive statistics], S4 [final models], and S5 [ *R^2^*]). Lastly, the internal validity of the final model was investigated by a bootstrapping validation procedure, creating 1000 random samples from the study population (see Supplementary Table S5).

### Qualitative methods

A purposeful sample of GPs was selected from different practices to participate in the semi-structured interviews to gain maximum insights to enrich and validate the quantitative findings. First, for each patient, the model-based MMCI was calculated, using the fixed effect of the patient and practice characteristics identified by the multilevel analysis. Next, for each practice, the difference between the mean calculated and the mean model-based MMCI for all patients within that practice was calculated. The five practices with the highest positive and the five practices with the highest negative differences were selected. One GP per practice was invited by one of the authors to participate and received written information concerning the study.

After the GP agreed to participate, the interviewer (the first author) scheduled an interview. At the start of the interview, the interviewer obtained oral informed consent. After a reminder, the response rate was 100% and all interviews were included in the analysis.

One author (the first author) conducted all semi-structured telephone interviews, for which the first author with two other authors had created a topic list. This list included a brief summary of the quantitative data. A topic list revision was considered after the analysis of the first three interviews, however, no revisions were needed. The interviewer avoided closed questions and encouraged participants to talk freely about their visions regarding factors that influence continuity of care in their practice during the observation period.

The interviews were conducted between 22 February 2021 and 17 March 2021 and took on average 17 min (range 13–31). All interviews were audiorecorded and transcribed verbatim. None of the participating GPs provided comments on their interview transcript. Afterwards, the interview data were coded and contact details for the GPs was deleted.

### Qualitative analysis

Two authors used thematic analysis according to Braun and Clarke to identify, analyse, and report patterns within the data.^46^ Thematic analysis allows for minimal organisation and detailed description of the data and may provide additional interpretation of various aspects of the research topic.^46^ To identify overarching key themes, the derived patterns were compared and discussed until consensus was reached.

## RESULTS

### Quantitative results

#### Descriptive analysis

The final dataset included 185 215 patients (Figure 1) who had 4 530 304 contacts with their practice, of which 2 734 776 contacts were with a GP between 2013 and 2018. The mean list size was 4027 patients per practice. The mean age of the patients was 40 years and the mean number of patients who were male was 43.3% (Table 1). The mean MMCI was lower in large practices (>4000 patients).

**Table 1.** Characteristics of the 48 general practices and their patients in 2013–2018

**Characteristic**	**Small practice,<2500 listed patients (*n*= 12)**	**Intermediate practice, 2500–4000 listed patients (*n*= 21)**	**Large practice, >4000 listed patients (*n*= 15)**	**All practices (*n*= 48)**
**Continuity indices per practice, MMCI, median (minimum–maximum)**	0.78 (0.68–0.87)	0.78 (0.63–0.90)	0.74 (0.57–0.79)	0.76 (0.57–0.90)

**Practice characteristics**				
List size, mean (SD)	2287.0 (204.9)	3141.0 (347.0)	6661.1 (1752.5)	4027.5 (2077.3)
Usual GP characteristics, mean (SD)				
Number of usual GPs	3.1 (1.1)	3.5 (1.3)	7.1 (1.8)	4.5 (2.3)
Number of working days/year	176.2 (63.3)	183.4 (47.9)	191.9 (30.0)	184.3 (48.0)
Number of GPs >5 years at practice	1.6 (1.0)	1.8 (0.6)	4.2 (2.0)	2.5 (1.7)
Locum GP characteristics, mean (SD)				
Number of locum GPs	6.8 (5.4)	5.7 (5.5)	5.7 (4.6)	6.0 (5.2)
Percentage of contacts with locum GPs	11.5 (8.0)	11.0 (9.0)	16.6 (14.9)	12.8 (11.0)
Number of employees, mean (SD)^a^	27.3 (13.7)	24.9 (16.5)	45.2 (11.9)	31.9 (17.0)
Training practice, *n* (%)^b^	4 (33.3)	14 (66.7)	13 (86.7)	31 (64.6)

**Patient characteristics^c^**				
Sex, male, %, mean (SD)	44.1 (4.2)	42.7 (3.6)	42.7 (3.6)	43.3 (3.3)
Age, years, %, mean (SD)^d^				
0–17	18.1 (2.8)	18.5 (5.3)	22.5 (7.4)	19.7 (5.8)
18–65	60.6 (5.2)	66.9 (6.1)	64.1 (5.7)	64.5 (6.1)
>65	21.3 (5.9)	14.6 (5.3)	13.4 (6.9)	15.9 (6.6)
Medical history, %, mean (SD)^e^				
>2 chronic diseases	35.6 (8.0)	30.4 (7.5)	30.2 (5.7)	31.6 (7.4)
Oncological disease	11.5 (2.7)	8.8 (3.3)	8.0 (2.9)	9.2 (3.3)
Coronary heart disease	5.3 (2.1)	4.4 (1.6)	4.4 (1.8)	4.6 (1.8)
Psychiatric disease	15.0 (3.2)	16.0 (3.2)	15.5 (3.0)	15.6 (3.1)
Income (area), %, mean (SD)^f^				
Low	22.0 (16.7)	29.9 (19.1)	40.0 (32.1)	31.1 (24.4)
Average	55.3 (14.7)	50.8 (26.0)	37.8 (30.8)	47.9 (25.9)
High	22.7 (17.2)	19.3 (26.9)	22.2 (53.5)	21.0 (25.7)
Migration background (area), %, mean (SD)^f^				
<10	39.3 (20.3)	29.9 (17.8)	13.7 (18.5)	27.6 (21.0)
10–30	39.9 (14.2)	46.2 (21.7)	34.7 (30.8)	41.5 (23.6)
30–70	16.8 (13.7)	24.0 (28.6)	51.6 (38.4)	30.9 (32.2)
Years since registration, mean (SD)^g^	12.7 (2.3)	11.1 (2.6)	9.8 (1.8)	11.1 (2.5)
Number of GP contacts per patient, mean (SD)	12.8 (2.7)	15.3 (1.9)	15.2 (2.2)	14.7 (2.6)
Type of GP contacts per practice, %, mean (SD)				
Telephone calls	16.4 (5.1)	20.5 (6.3)	19.9 (3.6)	19.2 (5.5)
Home visits	2.2 (0.7)	1.8 (1.0)	2.0 (1.5)	1.9 (1.0)
Face-to-face	80.7 (4.9)	76.5 (6.5)	77.5 (4.3)	78.0 (5.6)
Email	0.8 (1.5)	1.1 (1.5)	0.6 (1.0)	0.7 (1.3)

a *Excluding GPs.*

b *Trainee GP at practice.*

c *Determined at the patient level, aggregated at practice level.*

d *On 1 January 2016.*

e *Percentage of patients who have been diagnosed with chronic conditions (>1); cancer; coronary heart disease; or chronic psychiatric disorder.*

f *Estimated income and migration background based on national data on 1 January 2016; local income, corrected for differences between family compositions; and local migration background, that is, local percentage of population whose parents were born in Africa, South America, or Asia.*

g *On 31 December 2018. MMCI = Modified Modified Continuity Index. SD = standard deviation.*

#### Multivariate mixed-model analysis

Of the four continuity measures, the MMCI had the best fit, that is the highest explained variance in the final model with both practice and patient characteristics (*R^2^* 24.2%; see Supplementary Table S5).

An inverse, dose-dependent association was found between personal continuity and number of usual GPs, that is, the more usual GPs working in the practice, the more discontinuity (Table 2). The percentage of contacts with locum GPs had a similar association with personal continuity, that is, the higher the percentage of patient contacts with locum GPs the greater the discontinuity. List size, number of working days of the usual GPs, number of usual GPs working for >5 years at the practice, number of other employees, and being a training practice were not associated with personal continuity.

**Table 2.** Practice and patient characteristics associated with MMCI

**Characteristic**	**MMCI**		

	***ß***	**95% CI**	***P*-value**
**Practice characteristics**			
Number of usual GPs			<0.001
Q1 (2–3)	Reference	—	
Q2 (4–5)	−0.056	−0.091 to −0.021	
Q3 (6–7)	−0.062	−0.100 to −0.024	
Q4 (8–10)	−0.094	−0.135 to −0.053	
Percentage of contacts with locum GPs			<0.001
Q1 (0–4.2)	Reference	—	
Q2 (4.2–11.6)	−0.033	−0.071 to 0.006	
Q3 (12.7–18.6)	−0.075	−0.116 to −0.034	
Q4 (18.8–60.6)	−0.092	−0.131 to −0.052	

**Patient characteristics**			
Sex			0.001
Female	Reference	—	
Male	−0.003	−0.004 to −0.001	
Age, years^a^			<0.001
0–17	−0.042	−0.044 to −0.040	
18–65	Reference	—	
>65	0.027	0.024 to 0.030	
Medical history^b^			
Number of chronic diseases	0.005	0.004 to 0.005	<0.001
Psychiatric disease			<0.001
No	Reference	—	
Yes	0.023	0.021 to 0.026	
Oncological disease			<0.001
No	Reference	—	
Yes	0.007	0.004 to 0.011	
Coronary heart disease			<0.001
No	Reference	—	
Yes	−0.008	−0.013 to −0.004	
Days since registered^c^			<0.001
Q1 (0–1415)	Reference	—	
Q2 (1416–3197)	0.008	0.006 to 0.011	
Q3 (3198–6052)	0.013	0.010 to 0.015	
Q4 (5053–10 592)	0.017	0.014 to 0.019	
Contacts			
Number of contacts	0.004	0.004 to 0.004	<0.001
Percentage telephone calls	0.116	0.111 to 0.121	<0.001
Percentage home visits	0.061	0.050 to 0.072	<0.001

a *On 1 January 2016.*

b *Patients who have been diagnosed with chronic diseases.*

c *On 31 December 2018. MMCI = Modified Modified Continuity Index. Q = quartile. Q1 = 0%–25%. Q2 = 25%–50%. Q3 = 50%–75%. Q4 = 75%–100%.*

At the patient level, female sex was associated with higher personal continuity. Compared with younger adults (aged 18–65 years), patients who were aged >65 years had higher personal continuity. In contrast, children (aged <18 years) had lower personal continuity. Number of chronic diseases, psychiatric conditions, and oncological diseases were associated with higher personal continuity, whereas coronary heart disease was associated with lower personal continuity. A dose-dependent association was found between higher personal continuity and days since registration with the practice. Number of contacts, in particular the percentage of telephone calls and home visits, was associated with higher personal continuity. Area estimated income and migration background were not associated with personal continuity.

The confidence intervals of the 1000 bootstrap samples were narrow and the results were thus similar to those presented for the final MMCI model (see Supplementary Table S6).

### Interviews

**Figure 2.**
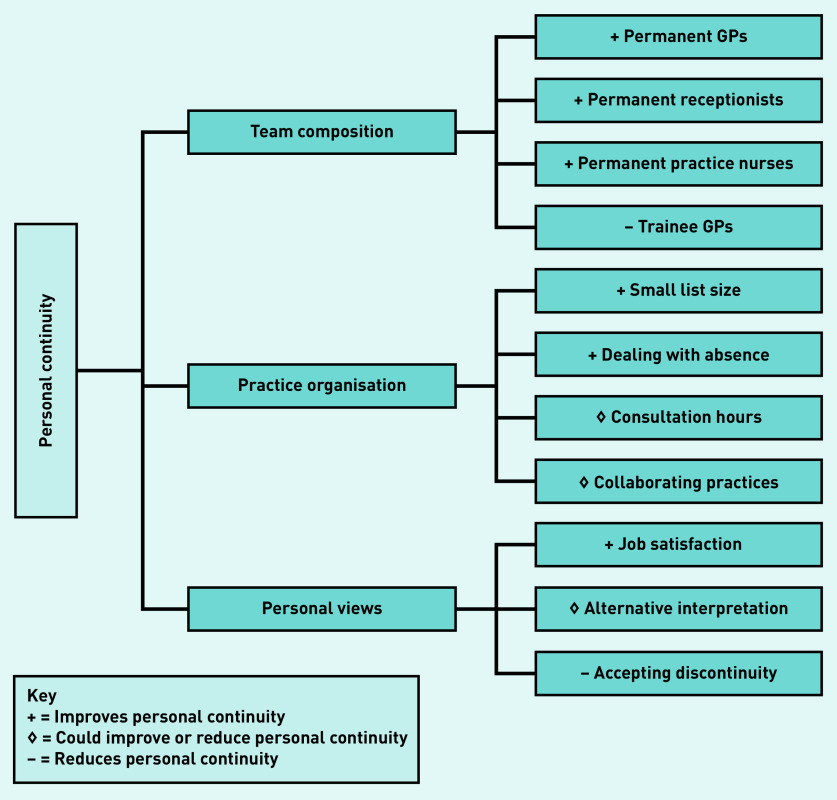
*Key themes influencing levels of personal continuity according to the GPs.*

Three overarching key themes were reported to affect personal continuity in these practices: team composition, practice organisation, and GPs’ personal views (Figure 2). Among the GPs that participated in the semi-structured interviews, 80% (*n* = 8/10) worked at a training practice (see Supplementary Table S3).

#### Team composition

All GPs considered the employment of a familiar team important in maintaining personal continuity:

*‘I’m sure we agree that part-time employment is incompatible with personal continuity, simply because something might happen on the day you’re not in.’*

(GP1, intermediate practice)

Some GPs said that they experienced difficulty in finding permanent colleagues, that is, who were available multiple days a week for months or years. According to those GPs, patients’ experience of personal continuity was also dependent on the familiar presence of other team members, including receptionists:

*‘When a patient calls, the telephone is answered by a familiar receptionist. My receptionists have been working with me for 40 years, so they know the patients for a long time. Because of our positive atmosphere, staff stays with us for decades.’*

(GP2, small practice)

According to one GP, the presence of GP trainees can threaten personal continuity:

*‘Every year, we have a different trainee* […] *who has to see complex patients as well.’*

(GP1, intermediate practice)

GPs expressed mixed views on the impact of practice nurses on personal continuity. They claimed that the presence of practice nurses was greatly appreciated because they provided continuity by coordinating care for patients with chronic diseases.^33^ Nevertheless, some GPs said that it could be difficult to keep an eye on certain vulnerable patients:

*‘During the study period, the number of practice nurses has increased. They have taken on a lot of interactions with the patients. Some of the care practice nurses provide occurs in addition to care provided by GPs, but a proportion of it substitutes for — and comes at the expense of — patients’ contacts with me or my* [GP] *colleagues.’*

(GP3, intermediate practice)

#### Practice organisation

According to the GPs, GPs working in practices with a small number of listed patients are expected to provide more personal continuity. However, those small practices had to hire locum GPs more often when the usual GP was unavailable:

*‘Fifteen years ago, we would record a taped message instructing the patients to call another practice whilst we were unavailable, for example during the holidays. This barrier was too high to overcome, so patients would wait. When the GP returned, their appointments would be jam-packed for three weeks* […] *Therefore, we hire locum GPs, many. We consider that phenomenal customer service, however, it does dilute personal continuity* … *’*

(GP3, intermediate practice)

Some large practices have organised themselves into multiple smaller practices. This way, they are able to cover each other’s shifts if the usual GP is absent. The planning of consultation hours was considered essential in terms of the availability of the usual GPs and, therefore, personal continuity:

*‘When I started working at our practice* [in 2017] *, I immediately cancelled the daily walk-in hours. It was impossible for patients to schedule an appointment with their named GP. Patients did not know in advance which doctor was on-call — they were unable to anticipate — so their consultation could be with a doctor they didn’t know.’*

(GP4, small practice)

The availability of the usual GP was often restricted. One GP described the differences between time consuming face-to-face consultations and time-efficient telephone consultations:

*‘We actually decided to increase the time allocated to face-to-face consultations. This way the consultation is less rushed, so I have time to talk about other things, for example, how their family is doing. To make this possible, we reduced the number of face-toface consultations and, of course, increased the number of telephone consultations* […] *For me, this change has been a great improvement. My patients appreciate this too.’*

(GP5, intermediate practice)

Another GP argued that the need for personal continuity was restricted to select patients, who should be prioritised in practice organisation:

*‘Young people often do not need to have a named GP, it does not matter to them as much. However, we actively allocate a named GP to people over 65 years, in order to improve the patient–doctor relationship, in anticipation of comorbidity that is expected within 10–15 years. Unless it is an emergency, of course.’*

(GP1, intermediate practice)

#### Personal views

As illustrated by the previous quotes, the interviewed GPs expressed their views on personal continuity directly or indirectly. Two GPs claimed that personal discontinuity is inevitable. Some GPs suggested alternative interpretations of personal continuity:

*‘* […] *Do you define continuity as “having a single healthcare provider, possibly with limited availability” or “having ample access to any* [general practice] *healthcare provider”? We offer the latter, not just for 46 weeks a year, but 52 weeks a year. You could say that our practice is never closed.’*

(GP3, intermediate practice)

*‘Perhaps we should define personal continuity as “I see one of these two doctors” rather than “I always see this specific GP”.’*

(GP1, intermediate practice)

Despite the practical challenges, the general view on personal continuity was optimistic:

*‘In my opinion, the familiarity between patients and* [general practice] *healthcare providers results in a very pleasant work environment.’*

(GP2, small practice)

## DISCUSSION

### Summary

The current study found that personal continuity was lower in a dose-dependent way when the number of usual GPs in a practice or percentage of contacts with locum GPs increased (highest versus lowest quartile −0.094 and −0.092, respectively, *P*<0.001). Being a training practice and list size were not associated with personal continuity. At the patient level, personal continuity dose-dependently increased when the patient had been registered for longer (highest versus lowest quartile +0.017, *P*<0.001). Personal continuity in these Dutch general practices was high (MMCI median 0.76, range 0.57–0.90), which is similar to that found in other studies.^32^

Qualitative interviews with GPs revealed three key themes affecting personal continuity: team composition, practice organisation, and GPs’ personal views. According to the GPs interviewed, a feasible way to increase personal continuity was working in small, stable, familiar teams with two to three usual GPs who share the workload and cover each other’s absences. Increasing the number of efficient telephone calls as opposed to time consuming face-to-face consultations, is in line with the quantitative finding that personal continuity was higher when the percentage of telephone consultations increases. Some GPs actively allocated older patients a named GP in anticipation of expected morbidities, which could explain the quantitative association between personal continuity and age. Being a GP training practice reduced personal continuity according to the GPs because the employment of trainees at a particular practice is temporary. However, no evidence to support this was found in the quantitative data in this study.

### Strengths and limitations

The major strength of this study was the mixed-methods design. The combined results provided complementary insights into the characteristics associated with personal continuity. This study was based on longitudinal real-world routinely collected data from 48 general practices covering all GP contacts over 6 years. The main outcome was based on contacts, registered by a particular GP working at a particular practice. In addition to practice characteristics, patient characteristics were included in the study; Dutch GPs are expected to record these routinely.^33^ Furthermore, because no international consensus exists on the best measure to calculate personal continuity in general practice, four different measures were included in this study. Another strength of this study was the in-depth thematic analysis of GP interviews. Although the participating GPs received the initial results for their practices, they were encouraged to share their views openly to avoid solely data-driven responses. No differences in GP responses between the included practices (that is, the five practices with the highest positive and the five practices with the highest negative differences) based on the sampling strategy were found.

A limitation of this study is that all practices were located in urban areas (the cities of Amsterdam and Haarlem). Therefore, the association between rurality and personal continuity as shown in previous studies^32^^,^^37^ has not been explored in the current study. However, the practices in this study varied in list size, patient population, and practice organisation, and had similar MMCI levels compared with other studies.^32^ The results are thus generalisable to Dutch general practices, in particular in urban areas. Furthermore, in the current study, access only to local estimations of migration background and income per patient were available, which may not correspond with the individual patient’s characteristics. This may have resulted in an underestimation of these associations with personal continuity. Finally, the current study focused on personal continuity between GPs and patients. According to the interviewed GPs, contacts with other healthcare providers (that is GP trainees and practice nurses) may contribute to perceived personal (team) continuity as well. Other types of continuity were not directly investigated (that is, management and information continuity).^1^

### Comparison with existing literature

To the authors’ knowledge, no other research has been published that has used mixed methods to study the association between practice and patient characteristics and the selected personal continuity measures. Guthrie (2002) and Palmer *et al* (2018), who studied both patient and practice characteristics, found an inverse association between large list size and personal continuity.^27^^,^^30^ In the current study, no association was found between list size and personal continuity. However, the number of GPs, which was associated with personal continuity, could be an indicator for list size. In contrast with the current study, both Guthrie and Palmer *et al* used a questionnaire to determine personal continuity. Guthrie also found that young males have lower odds of personal continuity than their female peers (odds ratio 0.86). A reversed association was observed with increased age, which Guthrie considered a ‘life cycle effect’.^27^ This could explain why female sex was associated with personal continuity (mean age 40 years) in the current study, whereas Coma *et al* (2021) found that males had higher personal continuity (mean age 49 years).^32^

Similar to this study, Coma *et al* studied various aggregated personal continuity measures at a practice level. They found a similar MMCI (0.73) with higher explained *R^2^* (56%), compared with the current study (0.76 and 24.2%, respectively). However, the majority of this *R^2^* in Coma *et al* was attributed to the single variable ‘Percentage of appointments booked with an assigned GP’ (39% in the variable-only model), which was not included in the current study. Therefore, it was anticipated that in the current study *R^2^* would be lower. Coma *et al* also found an inverse association between percentage of migration from a low-income country and MMCI (−0.14; *P*<0.05). The current study found no association between area estimates of migration background and personal continuity (*P* <0.001).

Based on the patients’ views described in a previous study, in the current study the authors had expected coronary heart disease to be associated with higher levels of personal continuity.^43^ However, in contrast to other chronic diseases, an inverse association was found. This could be explained by the increased employment of practice nurses, who provide care for patients with certain chronic diseases, including cardiovascular risk management.^33^ Additionally, patients with cardiovascular diseases may require urgent consultations more often, which therefore may involve a non-usual GP.^32^^,^^33^

Finally, Walker *et al* and Coma *et al* found that personal continuity in training practices was lower than that found for independently practising physicians, which is not in line with the findings in the current study.^32^^,^^39^ Forman *et al* suggested that awareness among GPs about the tension between providing continuity and educating young GPs may have resulted in team-based strategies to maintain continuity despite the presence of a trainee.^47^ The GPs who participated in the current study described this tension, so perhaps they had already implemented such strategies. This could explain why the current study found no statistically significant association between being a training practice and personal continuity.

### Implications for research and practice

Practice-level personal continuity is still high (MMCI 0.76). In the current study, MMCI was a better fit than the three other commonly used continuity measures (BBI, HI, and UPC). Complemented by GP’s views, suggestions to improve personal continuity include working in small teams with two to three usual GPs and pro-active allocation of older patients with chronic diseases to named GPs. In addition, patients and their families should be encouraged to schedule appointments with their usual GP to increase familiarity and mutual confidence. These changes may benefit patients and healthcare providers directly, because any improvement in personal continuity is associated with a lower use of out-of-hours services, fewer acute admissions to hospital, and lower mortality.^23^ Future research should evaluate the effect of the aforementioned suggestions (that is, working in small, familiar teams and informing patients about the potential benefits of personal continuity) as interventions. In addition, why of the four measures the MMCI had the best fit remains unclear, but will be part of future research by the present authors.

Differences in personal continuity between practices are partially explained by the included practice and patient characteristics. A large proportion of the explained variance is still unknown, although it may partially be explained by the qualitative findings in this study. For example, patients’ preference was not included, which may be dependent on confidence in their GP, the complexity of symptoms, or the convenience of the practice’s appointment system. Future studies should therefore incorporate patients’ views, by using a patient-reported outcome measure, and then compare those outcomes with the four continuity measures used in the current study.^48^

## Data

For privacy reasons the data cannot be made publicly available. Reasonable requests for conditional reuse of the data can be submitted to the corresponding author.
